# Subregional Hippocampal Morphology and Psychiatric Outcome in Adolescents Who Were Born Very Preterm and at Term

**DOI:** 10.1371/journal.pone.0130094

**Published:** 2015-06-19

**Authors:** James H. Cole, Maria Laura Filippetti, Matthew P. G. Allin, Muriel Walshe, Kie Woo Nam, Boris A. Gutman, Robin M. Murray, Larry Rifkin, Paul M. Thompson, Chiara Nosarti

**Affiliations:** 1 The Computational, Cognitive & Clinical Neuroimaging Laboratory, Department of Medicine, Imperial College London, Burlington Danes Building, Du Cane Road, London, United Kingdom; 2 Department of Psychosis Studies, Institute of Psychiatry, King’s Health Partners, King’s College London, De Crespigny Park, London, United Kingdom; 3 Imaging Genetics Center, University of Southern California, 4676 Admiralty Way, Marina del Rey, California, United States of America; University of Tuebingen Medical School, GERMANY

## Abstract

**Background:**

The hippocampus has been reported to be structurally and functionally altered as a sequel of very preterm birth (<33 weeks gestation), possibly due its vulnerability to hypoxic–ischemic damage in the neonatal period. We examined hippocampal volumes and subregional morphology in very preterm born individuals in mid- and late adolescence and their association with psychiatric outcome.

**Methods:**

Structural brain magnetic resonance images were acquired at two time points (*baseline* and *follow-up*) from 65 ex-preterm adolescents (mean age = 15.5 and 19.6 years) and 36 term-born controls (mean age=15.0 and 19.0 years). Hippocampal volumes and subregional morphometric differences were measured from manual tracings and with three-dimensional shape analysis. Psychiatric outcome was assessed with the Rutter Parents’ Scale at *baseline*, the General Health Questionnaire at *follow-up* and the Peters Delusional Inventory at both time points.

**Results:**

In contrast to previous studies we did not find significant difference in the cross-sectional or longitudinal hippocampal volumes between individuals born preterm and controls, despite preterm individual having significantly smaller whole brain volumes. Shape analysis at baseline revealed subregional deformations in 28% of total bilateral hippocampal surface, reflecting atrophy, in ex-preterm individuals compared to controls, and in 22% at follow-up. In ex-preterm individuals, longitudinal changes in hippocampal shape accounted for 11% of the total surface, while in controls they reached 20%. In the whole sample (both groups) larger right hippocampal volume and bilateral anterior surface deformations at *baseline* were associated with delusional ideation scores at follow-up.

**Conclusions:**

This study suggests a dynamic association between cross-sectional hippocampal volumes, longitudinal changes and surface deformations and psychosis proneness.

## Introduction

Very preterm birth (VPT; <33 weeks gestation) is associated with an increased risk of brain damage and consequent neurological disorders, neuropsychological, and behavioural impairments in childhood and later in life [1–5].

Long-lasting and widespread structural brain alterations have been described in VPT samples [2, 6, 7], suggesting that developmental changes in any brain region may result in a cascade of alterations in many other regions [8]. One of the areas of the brain consistently reported to be morphologically altered in VPT individuals is the hippocampus. This brain region is vulnerable to environmental influences implicated in the sequelae of very preterm birth, including hypoxic–ischemic damage [9], stress hormones [10], under-nutrition [11], and alteration of micronutrient supply [12].

Hippocampal volume decrements in VPT individuals compared to controls have been described in the first two decades of life, from infancy [13] to adolescence [11], as well as compromised hippocampal growth from infancy to school age [14]. At age 14 years we reported a 14% decrease in bilateral hippocampal volume in a VPT cohort, measured with a manual tracing technique, after adjusting for total brain volume [15]. Other authors have shown that hippocampal volumes of VPT infants are not disproportionally smaller compared to full-term controls relative to overall brain size [16] and that VPT infants’ hippocampal asymmetry is altered [17].

At a cognitive level, the hippocampus has been associated with processes involved in general intelligence [18], learning and memory [19], detection of novelty [20], forming semantic associations [21], and navigation [22]. At a behavioural level, structural alterations in hippocampus have been implicated in the pathophysiology of several psychiatric disorders including psychosis [23], post-traumatic stress disorder [24], bipolar affective disorder [25] and major depression [26]. Hippocampal volume abnormalities have been further described in individuals with an ‘at risk mental state’ for psychosis [27] and first-degree healthy relatives of individuals with schizophrenia [28], suggesting that hippocampal alterations may represent a critical intermediate disease phenotype.

Smaller hippocampal volumes in VPT born samples have been associated with low intelligence quotient [6] and with deficits in specific aspects of memory [11, 13, 29]. To our knowledge only one study to date has investigated the hippocampus in a VPT sample in relation to behavioural outcome and reported that smaller volume at age 2 was associated with increased hyperactivity and peer problems at age 5 [30]. We believe this is an area of priority given that several studies have described an association between very preterm birth and behavioural and psychiatric abnormalities in childhood and adolescence [31–35], and that many others have documented that individuals born VPT are at increased risk of developing major psychiatric disorders as adults [36–38].

In terms of development, the cytoarchitecture of the hippocampus is formed by the 34^th^ week of gestation [39]. However, neuronal proliferation in the hippocampus, in particular myelination, continues throughout adolescence and adulthood [39, 40]. Such changes may contribute to establishing the dynamic patterns of hippocampal maturation observed during the first decades of life [41]. Structural longitudinal hippocampal changes in adolescence following VPT birth have not yet been documented, although a few studies have described differential developmental changes in other brain areas in VPT born individuals compared to controls, with associated functional correlates [42–44]. Longitudinal changes in brain development are crucial to investigate, as there is evidence that trajectories of development rather than cross-sectional measures are stronger predictors of psychiatric and cognitive outcomes [45, 46].

Trajectories of development have described differential time-dependent courses for hippocampal subregions [41], which suggests that although the hippocampus is usually regarded a single brain region, it is structurally and functionally heterogeneous. The hippocampus is often divided along its anterior-posterior length into the head, body and tail and includes major subfields; the Cornu Ammonis area 1, 2, 3 (CA1, CA2, CA3), dentate gyrus and subiculum. These subfields have different afferent and efferent projections to other cortical areas [47] and may also have distinct functions in mediating learning [48] and affective regulation [49]. Novel neuroimaging analysis methods have been recently developed, which permit the identification of corresponding subregions across different brains [28, 50].

The primary aim of this study was to examine hippocampal volume and shape, which reflects subregional atrophy [50], in VPT individuals and controls in mid- and in late adolescence, and longitudinal changes between the two time points. The secondary aim was to investigate the association between hippocampal volume and shape and behavioural outcome. We hypothesised that at *baseline* (age 15 years), VPT individuals would have smaller hippocampal volumes than controls, based on our previous findings [15]. We expected group differences to be more pronounced in the anterior hippocampus, as selective memory deficits have been described in VPT samples [11]. A variety of memory processes are partly controlled by fronto-hippocampal networks [51], and the CA1 fields project directly to medial prefrontal cortices [52]. We further hypothesised that by *follow-up* (age 19 years) there would be diminished between-group differences in hippocampal volume and shape, on the basis of previous findings in other regions of interest (i.e., the corpus callosum) [44]. In terms of structure-function associations, we hypothesised that cross-sectional and longitudinal alterations of hippocampal volume and shape would be associated with a psychosis phenotype and specifically with mild forms of the expression of psychosis (i.e., delusional ideation), which are prevalent in the general population [53].

## Materials and Methods

### Participants

VPT participants were recruited from a cohort of individuals born before 33 weeks’ gestation between 1982 and 1984 who were admitted to the neonatal unit of University College London Hospital within 5 days of birth. From this population, 302 survived and were recruited as part of a long-term follow-up study [54–56]. At age 15 years 111 individuals received magnetic resonance imaging (MRI) [57]. At age 19 years, these individuals were re-contacted. Seventy-four (66%) were successfully scanned at both time-points. Preterm-born individuals who were not assessed did not differ significantly from those who were assessed in gestational age, Apgar scores at 1 and 5 minutes, gender, socio-economic status, or full-scale IQ at 15 years [43]. In this longitudinal study, analysis was restricted to those individuals who were assessed at both time points and also had completed the behavioural assessments of interest (n = 65, 58.5% of VPT individuals with *baseline* assessment).

A term-born comparison group of 71 individuals was recruited by advertisements in the press for the *baseline* assessment. Inclusion criteria were full-term birth (38–42 weeks) and birth weight >2500 grams. These same individuals were invited back for *follow-up* assessment. Successful MRI scanning was carried out in 36 (51%) individuals at both time-points. Exclusions criteria for both VPT individuals and controls were a history of neurological conditions including meningitis, head injury and cerebral infections, or any contra-indications to MRI scanning.

Participants were excluded from subsequent data analyses for the following reasons: one control participant had been diagnosed with depressive disorder, one VPT participant had cerebral palsy and two had severe hearing or visual impairment. The structural MRI scan for one VPT participant at *follow-up* could not be analysed due to signal artefacts. Thus, 61 preterm adolescents and 35 controls, none of which had received a clinical psychiatric diagnosis, were included in the data analysis. Data for an additional control participant was excluded from shape analysis due to processing error.

The Institute of Psychiatry, King’s College London Ethical Committee (Research) approved the study and the consent procedure used. Written informed consent was obtained from all participants at *follow-up* and from an accompanying parent at *baseline* assessment.

### Assessment of psychopathology

#### Baseline assessment

The Rutter Parents‘ Scale was used to assess emotional, attentional and conduct problems [58]. This scale was initially devised for screening purposes and is regarded as a valid instrument for studying psychopathology in unselected populations [59]. It is made up of 31 items, which are descriptions of behaviors, each of which is rated by a parent. The Rutter Parents’ Scale has been reported as having a sensitivity of 55% and a specificity of 94% [60].

The Peters Delusion Inventory (PDI) [61], a self-rating questionnaire, was used to measure a wide range of delusions, by investigating the distress, preoccupation and conviction with which a delusional belief is held. The PDI was created to assess lifetime delusional ideation and psychosis proneness in the general population. The PDI has good sensitivity and specificity, and its high negative predictive value support its usefulness as a psychosis proneness tool [62]. PDI scores were used as a continuous as well as a dichotomous variable, which classified ‘PDI cases’ (score 8) and ‘PDI non-cases’ (score<8) [62].

#### Follow-up assessment

We used a 12-item version of the General Health Questionnaire (GHQ-12) [63] to assess mental well-being in the domains of depression, anxiety, somatic symptoms and social withdrawal. A threshold of >4 and a conventional scoring (0,0,1,1) were used. If the GHQ is used as a dimensional model for psychological morbidity, GHQ score can be regarded as a proxy measure for the position of an individual on the underlying dimension without differentiating between 'cases' and 'non-cases'. The validity of this short version of the GHQ has been found to be as satisfactory as that of longer versions [64].

The PDI was administered again at *follow-up* assessment.

### MRI data acquisition

Magnetic resonance imaging was performed using a 1.5 Tesla system (General Electric Medical Systems, Milwaukee, WI). Three-dimensional T1-weighted spoiled gradient-echo (SPGR) sequences with 124 1.5 mm slices, in-plane resolution 1.5mm x 1.5mm, TR = 35 ms, effective TE = 5 ms, flip angle = 3 were acquired axially. The same image acquisition parameters were used on the same MRI scanner for both baseline and follow-up assessments.

### Whole brain analysis

Whole brain volume (WBV) at both time-points was calculated using Statistical Parametric Mapping software (SPM8, Wellcome Department of Imaging Neurosciences, University College London, UK). Whole brain volumes at *baseline* and *follow-up* assessments were used as covariates in subsequent analyses. In brief, SPGR images were masked to exclude non-brain tissues, and each voxel subsequently classified as grey matter or white matter, or other classes of tissue (e.g., CSF), by an automated segmentation algorithm. Total brain grey and white matter volumes were derived from the images in native space. Whole brain volume was calculated as the sum of total grey and white matter.

### Manual hippocampal segmentation

Pre-processing was conducted in accordance with the protocol established by Narr and colleagues [65]. For all T1-weighted images involved removal of non-cortical tissue, linear alignment to standard space, and reslicing in anterior commissure–posterior commissure (AC-PC) orientation. Bilateral hippocampi were traced manually using MultiTracer [66] with a well-established protocol [65] by a single trained rater blinded to diagnostic group (MLF). Inter-rater intraclass correlation coefficient (ICC) was calculated based on comparison with historical data from a panel of four separate raters who had previously been trained in use of the specific protocol. Inter-rater ICC = 0.90; while intra-rater ICC = 0.99, based on blinded repeat tracing of 6 hippocampal pairs. Boundaries of the hippocampus were delineated in the coronal plane, with simultaneous reference to sagittal and axial views. A single contour was traced on each contiguous slice, moving from anterior to posterior and inclusive of all hippocampal grey matter including the subiculum and thus incorporating the internal structures of the hippocampus, such as the dentate gyrus. The protocol dictated the exclusion of hippocampal white matter regions, such as the alveus and fimbria, so that the volumetric and shape measures reflected grey matter only. Hippocampal volumes were calculated by the sum of the areas measured from the centre of the first slice to the centre of the last slice, with a 1 mm sampling along the axis, with the square root of areas changing in a linear fashion from slice to slice.

### Three-dimensional hippocampal shape analysis

We used a Direct Hippocampal Mapping procedure [67], implemented in the Laboratory of Neuro Imaging (LONI) Pipeline software [68]. This automated procedure is based on the methods of Thompson and colleagues [50] and may be summarised as follows. A variational framework was used to define the direct mapping between two surfaces, in this case a standard neuroanatomical atlas of the hippocampus [69] and each subject’s hippocampus. Within this framework, Laplace-Beltrami eigen-features (i.e., linear operators that capture variance of the structure’s surface) were calculated to represent the hippocampus and capture the common geometry across surfaces. The atlas hippocampus was a triangulated mesh comprising 2000 vertices which was then mapped onto each subject’s hippocampal surface, giving spatial and statistical correspondence based on a given eigen-feature. For full details of the Direct Hippocampal Mapping analysis see the work by Shi and colleagues [67], for a illustrative description of the over-arching hippocampal shape mapping analysis, please refer to Figure two in Thompson et al., 2004 [50] and for details of the segmentation protocol please see Narr et al., 2004 [65].

### Statistical analysis

Statistical analysis of demographic and volumetric data was conducted using SPSS (v19.0, SPSS Inc., WI, USA). Cross-sectional and longitudinal analysis of demographic, cognitive and behavioural data between groups utilised χ^2^ tests (for gender, PDI and GHQ ‘caseness’), t-tests (age, GHQ-12, Rutter scale) and repeated measures ANOVAs (IQ and total PDI score). Correlation analyses between the two time points were performed for cognitive and behavioural variables. Volumetric analysis of hippocampal volume and WBV was carried out using a multivariate analysis of covariance for cross-sectional analysis at *baseline* and *follow-up*, with left and right hippocampal volume as the dependent variables, group as the experimental factor and sex as a covariate. As hippocampal volume and WBV are correlated [70], both raw hippocampal volume scores and ‘normalised’ scores were analysed, whereby hippocampal volume is divided by WBV (then multiplied by 1000, for convenience). As analyses using raw and ‘normalised’ values were similar, only those using normalized scores are reported here. Assessing the distribution of the data ascertained that PDI, Rutter scale and GHQ-12 scores were not normally distributed (Shapiro-Wilk tests p < 0.01). In order to make the data more appropriate for parametric statistical testing, these variables were log_10_ transformed, which reduced both skewness and kurtosis of the data (Shapiro-Wilk test p > 0.05).

Change between *baseline* (T1) and *follow-up* (T2) was quantified by using longitudinal change in WBV as an error term (WBV_error_ = WBV_T1_/WBV_T2_) [71]. This error term was used as a correction factor for longitudinal hippocampal change, which was calculated as follows:

$$Longitudinal\;change=\left(\left(\left(Volume_{T2}x\;WBV_{error}\right)\;-\;Volume_{T1}\right)/\;Volume_{T1}\right)\;x\;100$$

Paired-samples t-tests were used to test for within-group longitudinal changes in raw hippocampal volume and WBV. A repeated-measures ANCOVA was used to assess group effects on longitudinal change over time, with volume at *baseline* and *follow-up* (normalised for WBV_error_) being the within subject factor and group being the between-subject factor. Sex was the only covariate included in each ANCOVA despite the groups not being well-matched for age at scan nor IQ, as this would have been statistically inappropriate [72] as both were correlated with brain volumes. The False Discovery Rate (FDR) [73] correction was used to correct for the multiple comparisons carried out, using q = 0.05. For the between-group volumetric analysis correction was conducted to account for six regions (i.e. left and right hippocampus and WBV) and time-point (i.e. baseline and follow-up). For within-group pairwise analysis the three volumetric measures were corrected for within each group.

Associations between cross-sectional and longitudinal normalised hippocampal measures and clinical measures (birth weight and gestational length), behavioural/psychiatric outcomes at *baseline* (Rutter Parents’ Scale and PDI scores) and at *follow-up* (GHQ-12 and PDI scores) were explored using a multiple linear regression approach. Again FDR correction with q = 0.05 was carried out, this time correcting for the five clinical or behavioural outcome variables tested.

The feature selected for three-dimensional hippocampal shape analysis was the tail-to-head trend of the hippocampus, which is represented by a graph comprised of a node at the centroid of each contour, thus giving a measure of the distance between the medial core and each surface vertices of the structure. This measure is analogous to that used in our previous work [26], but is entirely intrinsic and cannot be influenced by variations in translation or rotation between structures [66]. This approach captures the degree to which the atlas hippocampus was deformed in order to match each subject’s hippocampus, either as an expansion or contraction, at each surface vertex. Furthermore, statistical maps for hippocampal surface-related measures were generated in stereotaxic space to account for differences in baseline brain size that may be associated with demographic variables such as sex or age [50]. To quantify group differences (VPT adolescents and controls), two-tailed t-tests were conducted at each vertex, to compare hippocampal deformations (i.e. expansions or contractions) cross-sectionally at *baseline* and *follow-up* assessments, as well as longitudinally within each group. To correct for the multiple comparisons across the 2000 atlas vertices, 50,000 permutations were run by randomising group membership and comparing significance maps of real and randomised groups. This process generated a corrected p-map that represents statistical differences in hippocampal shape between groups. Three-dimensional hippocampal shape was further investigated in relation to group and PDI ‘caseness’, as our previous work demonstrated selective associations between behavioural outcome and regional brain morphology (e.g. hyperactivity scores and caudate volume) in VPT-born individuals but not in controls [74].

## Results

### Demographic, cognitive and behavioural data

Mean gestational age for the VPT group was 28.8 weeks (n = 61, SD = 2.2), for controls it was 40.0 weeks (n = 31, SD = 1.4); mean birth weight for the VPT group was 1248.3 grams (n = 61, SD = 380.2), for controls it was 3250.3 grams (n = 33, SD = 388.5).

Demographic, cognitive and behavioural data for VPT adolescents and controls at *baseline* and *follow-up* are shown in Table 1. There was no between-group difference in the length of time between *baseline* and *follow-up assessments* (*p =* 0.40). At both *baseline* and *follow-up* VPT adolescents were significantly older and had lower IQ score than controls. IQ scores at *baseline* and *follow-up* were significantly correlated (r = 0.69, *p <* 0.001).

**Table 1:** Demographic, cognitive, behavioural data and raw hippocampal volumes in mm3 for preterm adolescents and controls at baseline and follow-up.

	Baseline (age 15 years)		Follow-up (age 19 years)		Test statistics
	Preterm (n = 61)	Control (n = 35)	Preterm (n = 61)	Control (n = 35)	
Age, years*	15.46 0.45	14.99 0.73	19.61 0.86	18.97 0.82	Baseline t = 4.94 Follow-up t = 5.55
**Sex (male/female)**	32/29	18/16	32/29	18/16	Baseline X^2^ = 0.03 Follow-up X^2^ = 0.01
**Full-scale IQ** ^**1**^ ^**,**^ ^**3**^	99.24 15.70	108.15 12.10	96.58 13.68	102.83 12.49	Group F = 4.23 Time F = 3.42 Group*Time F = 0.29
**Verbal IQ** ^**1**^ ^**,**^ ^**2**^	97.86 14.93	105.94 10.12	93.97 15.82	99.86 12.50	Group F = 4.26 Time F = 5.63 Group*Time F = 0.24.
**Performance IQ**	100.6 18.02	108.54 15.24	99.45 15.06	103.43 15.68	Group F = 1.38 Time F = 1.41 Group*Time F = 0.62
**Rutter Total Score**	6.97 7.58	8.00 6.32	-	-	Baseline t = 0.65
**PDI total** ^§^	5.16 4.65	5.34 3.80	7.41 7.35	7.18 6.37	Group F = 0.047 Time F = 2.08 Group*Time F = 0.82
**PDI caseness (8) %**	27.9%	22.9%	34.4%	42.9%	Baseline X^2^ = 0.26 Follow-up X^2^ = 0.67
**GHQ total**	-	-	1.17 2.20	1.23 2.29	Follow-up t = -0.12
**GHQ caseness (>4) %**	-	-	6.6%	5.7%	Follow-up X^2^ = 0.03
**Left hippocampus** ^**2**^	2389.7 427.7	2409.5 435.5	2595.7 403.0	2582.5 396.6	Group F = 2.23 Time F = 10.54 Group*Time F = 0.07
**Right hippocampus** ^**2**^	2199.7 364.8	2311.6 338.6	2415.9 449.5	2397.3 374.1	Group F = 2.81 Time F = 8.96 Group*Time F = 0.26.

Values reported in mean (standard deviation) format. PDI = Peters Delusions Inventory.

* VPT participants were older than controls at both *baseline* and *follow-up assessments* (p < 0.001) from t-tests

^1^ Significant group effect (VPT individuals vs. controls) (*p* < 0.05) from repeated measures ANOVA

^2^ Significant effect of time (baseline vs. follow-up) (*p* < 0.05) from repeated measures ANOVA

^3^ Significant interaction between group (VPT individuals and controls) and time (baseline vs. follow-up) (*p <* 0.05) from repeated measures ANOVA.

^**§**^ At *baseline* (age 15 years) PDI data were missing for 18 VPT born participants; at *follow-up* (age 19 years), for 7 VPT born participants and one control.

At *baseline*, behavioural/psychiatric scores did not differ significantly between the two groups (e.g. Rutter total score, PDI total, PDI caseness). At *follow-up* assessment, results were similar and no statistically significant between-group differences were detected (e.g. GHQ total, GHQ caseness, PDI total, PDI caseness). Correlations between Rutter Total scores and PDI scores at *baseline* and between GHQ-12 scores and PDI scores at *follow-up* were non-significant. A significant correlation was observed between PDI total scores at *baseline* and *follow-up* assessments (n = 43, r = 0.47, *p <* 0.001).

### Cross-sectional neuroimaging data

#### Volumetric analysis

Baseline assessment: There was no significant difference in left (p = 0.62) or right ‘normalised’ hippocampal volume (*p =* 0.55) between VPT individuals and controls, after adjusting for sex. The preterm group had significantly smaller WBV compared to controls (t = 4.11, *p <* 0.001).

Follow-up assessment: There was no significant difference in ‘normalised’ left (*p =* 0.26) or right hippocampal volume (*p =* 0.29) between VPT individuals and controls. Again, the VPT group had reduced WBV compared to controls (t = 4.46, *p <* 0.001). Descriptive statistics for bilateral hippocampal volumes in VPT adolescents and controls are given in Table 1. P-values are all reported after FDR adjustment for multiple comparisons.

#### Shape analysis

**Fig 1:**
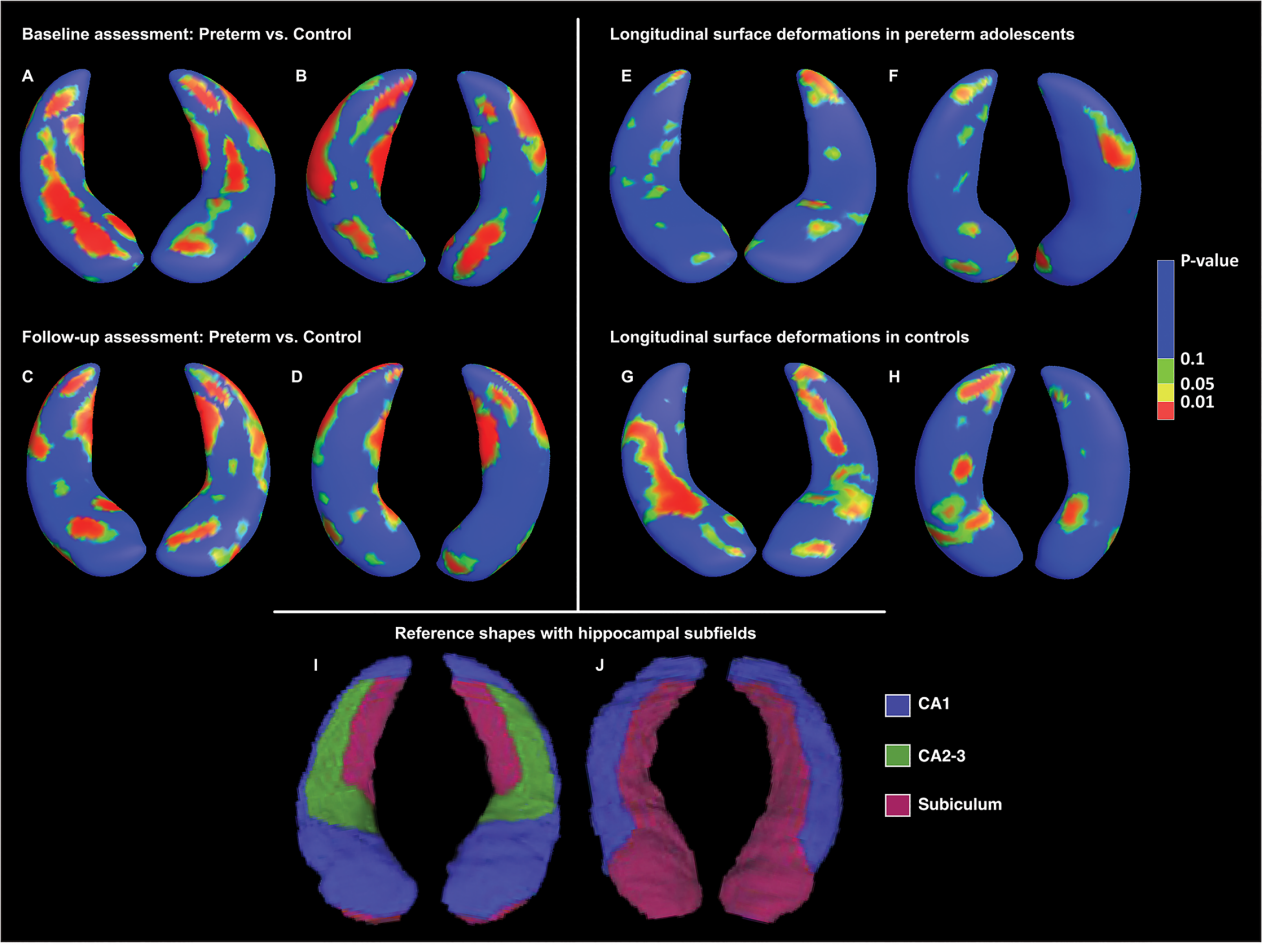
Shape analysis showing cross-sectional and longitudinal surface deformations in preterm adolescents compared to controls. P-values denote statistical significance of control > preterm across 2000 surface vertices, corrected for multiple tests (50,000 permutations). (A) Baseline comparison, superior view, with left hippocampus on the right. (B) Baseline comparison, inferior view with the left hippocampus on the left. (C) Follow-up comparison, superior view, with left hippocampus on the right. (D) Follow-up comparison, inferior view with the left hippocampus on the left. (E) Longitudinal surface deformations preterm adolescents, superior view, with left hippocampus on the right. (F) Longitudinal surface deformations in preterm adolescents, inferior view with the left hippocampus on the left. (G) Longitudinal surface deformations in the control group, superior view, with left hippocampus on the right. (H) Longitudinal surface deformations in the control group, inferior view with the left hippocampus on the left. (I) Superior view of representative hippocampal shapes with approximate subfields labelled. CA1 = blue, CA2-3 = green, subiculum = mauve. (J) Corresponding inferior view with subfields labelled.

Baseline assessment: There were significant surface deformations in the ex-preterm adolescents compared to controls, for both the left and right hippocampus. Several clusters of surface contractions were found, on both posterior and anterior portions of the structure (in the subiculum and CA1 subfield extending into the CA2-3 subfields), equating to approximately 28% of the surface of left and right hippocampi (see Fig 1A and 1B and Table 2). There were no areas where significant expansions in the VPT group compared to the controls were evident.

**Table 2:** Cross-sectional hippocampal shape analysis details

Contrast	Number of vertices with p < 0.05	Percentage hippocampal difference	Anatomical location
**Baseline left**	562	28.10%	Subiculum, CA1, CA2, CA3
**Baseline right**	568	28.40%	Subiculum, CA1, CA2, CA3
**Follow-up left**	423	21.15%	Subiculum, CA1
**Follow-up right**	468	23.40%	Subiculum, CA1

Based on 2000 vertices per hippocampus.

Follow-up assessment: There were still substantial numbers of significant surface contractions in the preterm group. However, there was a decrease in the number of surface points differing between groups and the deformations were now almost entirely concentrated in the tail of the left and right hippocampi (equating to the subiculum and CA1 subfield), while hippocampal head and body were no longer significantly different (see Fig 1C and 1D and Table 2).

### Longitudinal neuroimaging data

#### Volumetric analysis

Within-group analysis in ex-preterm adolescents indicated that left hippocampal volume increased 13.9% between *baseline* and *follow-up* and that right hippocampal volume increased 14.7%. Both increases were statistically significant after FDR correction (left, t = 4.42, *p <* 0.001; right, t = 4.14, *p <* 0.001). In controls left hippocampal volume increased significantly by a magnitude of 12.5% (t = 2.86, *p =* 0.016), but the 8.2% increase in right hippocampal volume was not significant (*p =* 0.19). Mean longitudinal hippocampal increases were calculated using WBV_error_ to correct for measurement error over time [71]. Repeated measures ANCOVA indicated that there were no group effects on longitudinal change for ‘normalised’ bilateral hippocampal volumes (all *p*>0.05). Furthermore, the effect of time on normalised bilateral hippocampal volumes was not significant across groups (all *p*>0.05). There were no longitudinal changes in WBV or in intracranial volume for either group.

#### Shape analysis

When comparing longitudinal changes in hippocampal shape within each group, there were minor expansions in both left and right hippocampus in the VPT group, predominantly in the tail of the structure and accounting for approximately 10% of the total surface (see Fig 1E and 1F and Table 3). In controls however, considerably greater surface expansions were found over time, particularly in the left hippocampus, where over 25% of the surface had expanded. The expansions in the left were mainly in the hippocampal tail, whereas the expansions in the right were found in the mid-section (see Fig 1G and 1H and Table 3).

**Table 3:** Longitudinal hippocampal shape analysis details.

Contrast	Number of vertices with p < 0.05	Percentage hippocampal difference	Anatomical location
**Preterm left**	213	10.65%	Subiculum, CA1
**Preterm right**	218	10.90%	Subiculum, CA1
**Controls left**	517	25.85%	CA1, CA2, CA3
**Controls right**	317	15.85%	CA2, CA3

Based on 2000 vertices per hippocampus.

### Associations between neuroimaging, behavioural/psychiatric and neonatal data

#### Volumetric analysis

Baseline assessment: Linear regression analyses were performed to assess the association between bilateral hippocampal volumes (normalised for WBV) and outcome measures in the whole sample (VPT participants and controls), as well as within group. Bilateral normalised hippocampal volumes were not significantly associated with PDI and Rutter scores.

Gestational age and birth weight were not significantly associated with cross-sectional bilateral hippocampal volumes in the VPT group (all p > 0.05).

**Fig 2:**
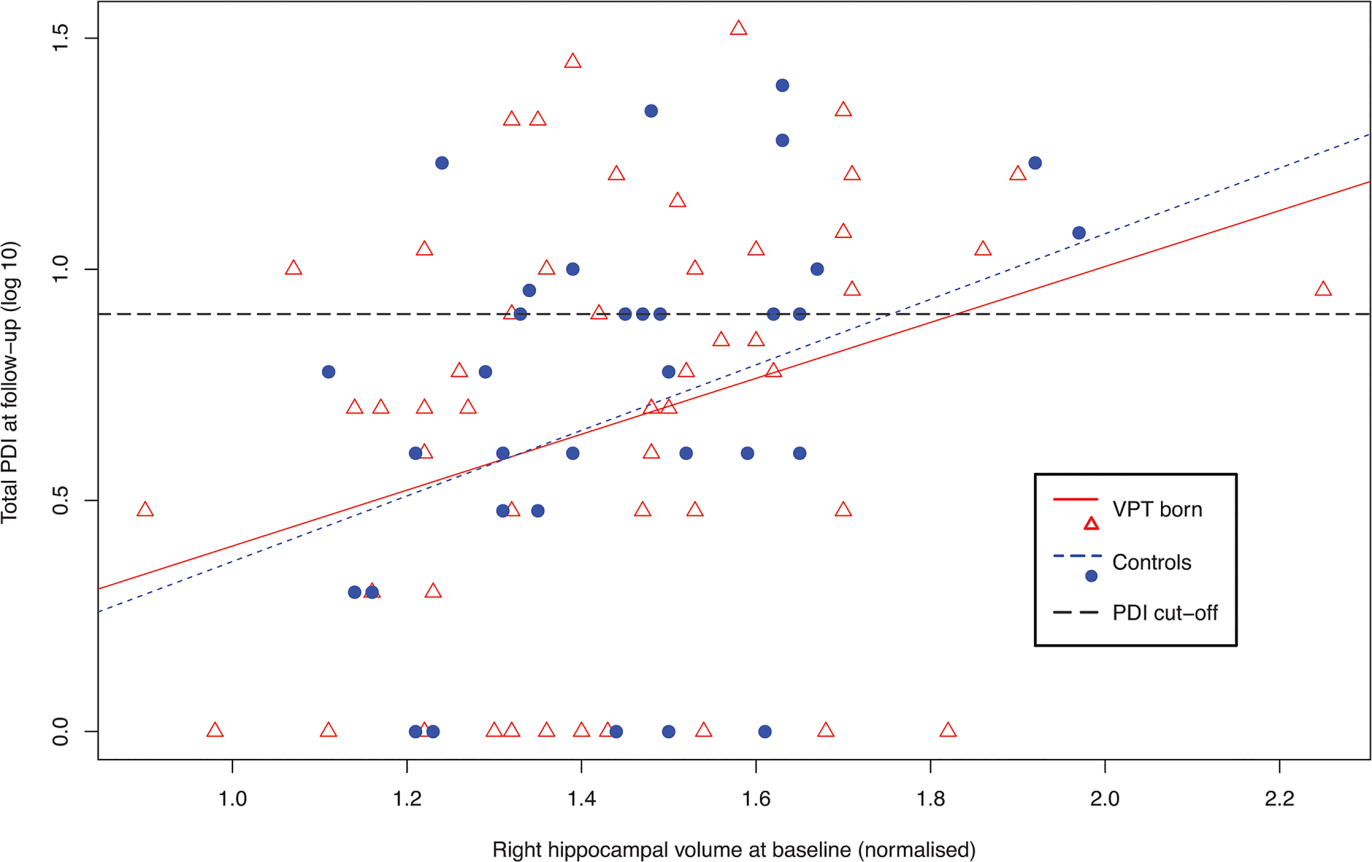
The relationship between baseline right hippocampal volume and PDI score at follow-up. The plotted values are based on the normalised right hippocampal volume at baseline and the log10 transformed total PDI score at follow-up. This relationship was the sole significant association between imaging and behavioural measures in the analysis, after FDR correction for multiple testing (p = 0.032). R^2^ (ratio of the sum of squares explained by the regression model and the total sum of squares around the mean) of right hippocampus at age 15 years: VPT = 0.11, controls = 0.12. The horizontal grey line represents the cut-off in terms of PDI ‘caseness’, defined at follow-up using PDI ≥ 8, which after log10 conversion = 0.9031. Plotted points that lie on the grey are defined as PDI cases.

Follow-up assessment: Right and left normalised hippocampal volumes were not significantly associated with PDI scores. However, baseline right hippocampal volume was significantly positively associated with PDI total score at *follow-up* in the whole sample (see Table 4 and Fig 2) as well as within group (VPT: R^2^ = 0.11 *=* 0.18, *p* = 0.016; control: R^2^ = 0.12, *=* 0.17, *p =* 0.04). Furthermore, those individuals who scored as a PDI ‘case’ at follow-up had larger normalised right hippocampal volume at *baseline* than those scoring as ‘non-case’ (F = 15.0, *p <* 0.001). Left hippocampal volume at *baseline* was not significantly associated with PDI scores at *follow-up*. Bilateral normalised hippocampal volumes were not significantly associated with continuous or dichotomous GHQ scores (all *p* > 0.05).

**Table 4:** Results of linear regression analyses with behavioural scores as dependent variables and cross-sectional and longitudinal normalised hippocampal volume (all participants).

Predictors (Behavioural and clinical measures)		Outcome variables					
		Baseline hippocampal volume		Follow-up hippocampal volume		Longitudinal change in hippocampal volume	
		Left	Right	Left	Right	Left	Right
**Clinical**	**Birth weight**	β = 0.0; t = 1.09; p = 1.00	β = 0.0; t = 1.62; p = 0.44	β = 0.0; t = 1.13; p = 0.67	β = 0.0; t = 1.21; p = 0.89	β = -0.023; t = -0.19; p = 1.00	β = -0.027; t = 0.13; p = 1.00
	**Gestational length**	β = -0.023; t = -1.32; p = 0.95	β = -0.023; t = -1.62; p = 0.44	β = -0.02; t = -1.23; p = 0.67	β = -0.022; t = -1.23; p = 0.89	β = 8.23; t = 0.36; p = 1.00	β = 4.54; t = 24.25; p = 1.00
**Baseline**	**PDI total**	β = -0.08; t = -0.55; p = 0.95	β = 0.10; t = 0.83; p = 0.451	β = 0.05; t = 0.58; p = 0.57	β = 0.13; t = 1.34; p = 0.44	β = 37.55; t = 0.29; p = 0.77	β = 63.07; t = 0.46; p = 0.65
	**Rutter scale total**	β = 0.043; t = 0.53; p = 1.00	β = -0.034; t = -0.52; p = 0.602	β = -0.047; t = -0.62; p = 0.67	β = -0.077; t = -0.95; p = 0.89	β = -93.90; t = -0.89; p = 1.00	β = -26.42; t = 110.86; p = 1.00
**Follow-up**	**PDI total**	β = 0.083; t = 1.09; p = 1.00	β = 0.17; t = 2.81; p = 0.032*	β = 0.13; t = 1.90; p = 0.31	β = 0.085; t = 1.14; p = 0.89	β = 90.28; t = 0.92; p = 1.00	β = -111.96; t = 103.18; p = 1.00
	**GHQ-12**	β = -0.067; t = -0.25; p = 1.00	β = 0.28; t = 1.31; p = 0.44	β = 0.35; t = 1.45; p = 0.61	β = 0.47; t = 1.79; p = 0.39	β = 488.21; t = 1.42; p = 1.00	β = 154.02; t = 360.32; p = 1.00
**Model statistics**	**Intercept**	2.02	2.25	2.05	1.97	-109.00	181.98
	**Standard error**	0.28	0.23	0.25	0.27	359.30	377.30
	**R** ^**2**^	0.04	0.18	0.10	0.09	0.05	0.02
	**p-value**	0.73	0.048	0.20	0.25	0.59	0.93

Behavioural variables (PDI, Rutter scale, GHQ-12) were all log_10_ transformed prior to statistical analysis. Results are derived from multiple linear regression analysis and are reported in this form: β-coefficient; t-value; FDR adjusted p-value for each coefficient.

* Denotes statistically significant result after FDR correction [73] for multiple testing.

Longitudinal hippocampal volume change: Volumetric bilateral hippocampal changes between the two time-points were not significantly associated with PDI scores, Rutter scores, GHQ scores, or birth weight and gestational length (all FDR adjusted p > 0.05). (see Table 4).

There were no significant associations between continuous or dichotomous GHQ scores and cross-sectional and longitudinal bilateral hippocampal volumes and in the whole sample and within group (all *p*>0.05).

### Shape analysis

**Fig 3:**
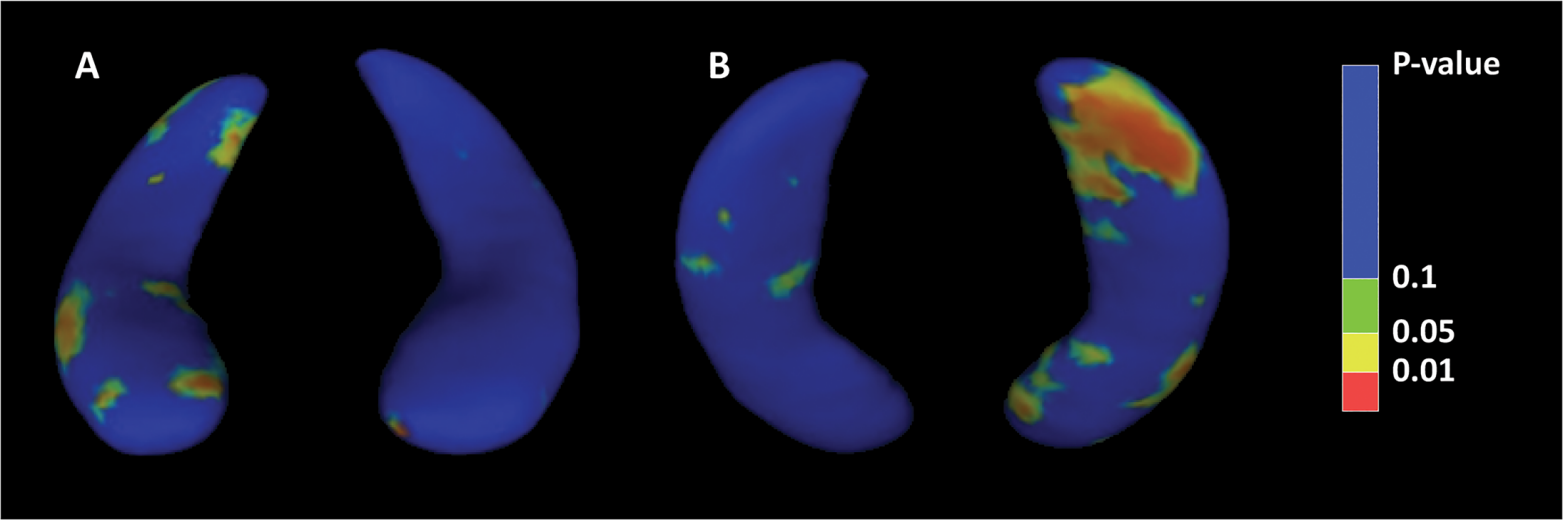
Longitudinal shape analysis showing surface deformations in ‘PDI-cases’ compared to ‘PDI-non-cases’. P-values denote statistical significance of follow-up > baseline across 2000 surface vertices, corrected for multiple tests (50,000 permutations). (A) Superior view, with right hippocampus on the left. (B) Inferior view, with right hippocampus on the right.

As right hippocampal volume at *baseline* was significantly associated with PDI total score, surface deformations were also investigated in relation to PDI caseness, defined at follow-up. A dichotomous rather than a correlational approach was chose in order to maximise statistical power. After permutation correction, results for the right hippocampus were statistically significant (*p =* 0.005) in the whole sample (VPT individuals and controls), whereby PDI ‘cases’ showed surface deformations compared to PDI ‘non-cases’, especially in the subiculum and CA1 subfield (Fig 3). Within group analyses did not show statistically significant results.

## Discussion

Although total hippocampal volumes did not differ between VPT adolescents and controls at both 15 and at 19 years of age, there were extensive differences in hippocampal shape between the groups. At *baseline*, localized hippocampal subregional deformations were noted across approximately 28% of the total hippocampal surface in the VPT group compared to controls, especially in the posterior and anterior portions of the structure, in the subiculum and CA1 subfield extending into the CA2-3 subfields. At *follow-up*, deformations were almost entirely concentrated bilaterally in the hippocampal tail, subiculum and CA1 subfield.

The shape of a brain structure has been hypothesized to be determined by the physical properties of neural tissue and by patterns of neural connectivity [75], which have been shown to be altered following preterm birth [76]. These results differ from our previous findings in a similar group of VPT adolescents at 14 years, which revealed a 14% lower bilateral hippocampal volume compared to controls [15]. The use of different subject samples, hippocampal measurement techniques and definition of hippocampal boundaries may partly explain this inconsistency.

The current results showed that volumetric hippocampal increases between mid- to late adolescence were comparable between the groups, in contrast to findings of altered hippocampal growth trajectories in childhood [14] and differential changes in the volume of the cerebellum [43] and in the mid-sagittal area of the corpus callosum [44] that we previously reported in the same cohort between the same time points. Significant age-related changes in total hippocampal volume in normative samples have not been found between 4 and 25 years of age [41] and between 16 and 65 years [77], although a recent study showed a negative correlation between bilateral hippocampal volume and age in healthy 12 to 24 year olds [78]. As far as we are aware, this is the first study to investigate hippocampal volume changes in a relatively short time span during adolescence, and in the absence of specific training interventions [79]. Studies of different age groups are difficult to compare. Moreover, when comparing longitudinal changes in hippocampal shape within each group, there were minor expansions in both left and right hippocampus in the VPT group, predominantly in the tail of the structure and accounting for approximately 10% of the total surface. Conversely in controls considerably greater surface expansions were found over time, particularly in the left hippocampus, where over 25% of the surface had expanded. The left hippocampal expansions were mainly in the tail region, whereas the expansions in the right were found in the hippocampal body, in the vicinity of the CA1 subfield. Lateralised dynamic changes in hippocampal subdivisions have been reported by others [41], but the reasons for these lateralised effects remain unclear. Significant shape change difference between infancy to school-age between VPT children and controls were not observed by Thompson and colleagues (although hippocampal growth was reduced in VPT children) [14], suggesting that selective hippocampal subregions may be particularly vulnerable to late maturational alterations following VPT, possibly as a consequence of earlier alterations growth trajectories.

When investigating hippocampal volume in relation to psychiatric outcomes, we found that right hippocampal volume at *baseline* was positively associated with delusional ideation scores at *follow-up*. This was the case in all subjects, VPT individuals and controls, who also had comparable PDI mean scores and number of PDI ‘cases’. The hippocampus has been previously implicated in affective, social and mnemonic processing [80–82]. Alterations of these processes may contribute to the development of delusional ideation. For instance, a study found that scores on the Peters’ Delusional Inventory were associated with self-reported measures of memory errors [83]. Furthermore, individuals with high levels of delusional ideation show a tendency to ‘jump to conclusions’ when asked to make decisions under uncertainty [84]. This cognitive bias has been put forward as a possible mechanism of delusion formation and maintenance [85] and has been associated with impaired working memory [86]. The hippocampus plays a central role in a variety of tasks involving working memory [87], and in preterm born children working memory deficits have been associated with neonatal hippocampal volume [13].

*Greater* hippocampal volume at age 15 years was associated with delusional ideation scores at age 19. These results appear at first counterintuitive, as decreased hippocampal volume has been described in individuals experiencing delusional thinking—in schizophrenia [88] and in major depressive disorder [26]. However, enlarged hippocampal volumes have been described in those individuals at high risk of developing schizophrenia [27], in adolescents with autism [89] and in those with attention-deficit hyperactivity disorder [90]. Normative data have demonstrated that the hippocampus follows a dynamic developmental trajectory, with regional hippocampal increases and decreases during the first two decades of life [41]. We speculate that our findings may be explained by alterations in these dynamic developmental changes, that sub-clinical psychotic problems may be differentially associated with hippocampal volumes depending on the stage of development, or that an enlarged hippocampus may be a precursor of sub-clinical psychiatric problems such as delusional ideation. One caveat to highlight when interpreting these results is that the amount of variance in hippocampal volume explained by PDI score was low (5% for VPT individuals, 10% for controls), thus there are clearly other behavioral and genetic factors that will influence hippocampal development during adolescence, that we did not capture in our study. Moreover, the relationship between change in hippocampal volume and total PDI score was not statistically significant after FDR correction. This may be due to a relative stabilization of trajectories within individuals when looking across the range of PDI scores. Alternatively, it may be that the behavioral and neuroimaging measures are both sufficiently noisy to mask any underlying relationship; such relationships are seldom reported in the longitudinal neuroimaging literature. For this reason, it is anticipated that larger samples and more precise measures, both for measuring delusional ideation and hippocampal structure, will be needed to establish if aberrant brain development during adolescence is related to the presence of delusions.

Right hippocampal shape deformations at *baseline*, mainly localized in the anterior portion of the region, were observed in individuals who scored as a PDI ‘case’ at *follow-up* compared to those who scored as a ‘non case’ (VPT individuals and controls). No statistically significant association between hippocampal shape at *follow-up* and PDI scores were found. Distinct areas of the prefrontal cortex receive projections from neurons in the head of the hippocampus, therefore it could be speculated that these results support the idea that psychiatric disorders characterized by delusional beliefs such as schizophrenia involve alterations in fronto-temporal circuitry [91]. Variations in hippocampal morphology and shape, preferentially affecting the anterior of the structure, have been reported in neuropsychiatric disorders with neurodevelopmental components such as schizophrenia [92, 93] and structural abnormalities of subicular dendrites have further been reported in individuals with schizophrenia and mood disorders [94]. The lack of observed associations between hippocampal shape at *follow-up* and behavioral scores collected at the same time-point may be explained by the fact that shape differences were then mostly observed in the posterior hippocampal segment.

Limitations of this study include the limited sample size, and the fact that the neurodevelopmental profile of VPT individuals who were born in the 1980s may differ from that of younger cohorts who may have received more advanced forms of neonatal care. Regarding the study methodology, a potential problem to the interpretation of morphological analysis is its limited anatomical accuracy. To date, surface shape-based such as the method we used, provides the most valid way of investigating changes *in vivo*, but they do not achieve the precision obtained by the study of neuropathological data, which allows assessment at a microscopic level and can differentiate between the outer (e.g. CA1, subiculum) and inner (e.g. dentate gyrus) subfields. Furthermore, only one rater conducted the hippocampal segmentation. Ideally multiple raters would be available to increase the robustness of the segmentations and to allow testing of how different raters may influence results. Nevertheless the rater in the current study achieved high reliability with a historical dataset on which the protocol was defined as well as excellent intra-rater reliability, meaning that additional raters are unlikely to have greatly influenced the current findings. Another limitation of the study may be the representativeness of our control sample, a large proportion of whom scored as a PDI ‘case’ at *baseline* (23%) and *follow-up assessments* (43%). Psychotic experiences and beliefs have been reported in the general population with prevalence up to 40% [95]. However, the positive predictive value of the PDI has been estimated to be rather low (28%), which is a possible reflection of the low rate of the psychiatric disorders with psychotic features in non-clinical samples [62]. The ex-preterm and control groups were not well matched for age at scan or IQ, which may potentially complicate the interpretation of the results, as the neuroanatomical expression of psychiatric disorders in young populations is dynamic [96]. Finally, we did not detect any sex-specific effects in our study, despite the growing evidence that sexual dimorphism is important after preterm birth [5], something which could be explore more explicitly in future research.

To conclude, although this study did not reveal significant differences in hippocampal volume between VPT individuals and controls in mid- and late adolescence, a substantial percentage of the total hippocampal surface showed subregional deformations in the VPT group, suggestive of atrophy, predominantly in the posterior and anterior regions. Cross-sectional and longitudinal hippocampal volumes were dynamically associated with delusional ideation scores in young adulthood. These results further our understanding of the structural correlates underlying long-term neurodevelopmental consequences of very preterm birth. They also support the idea of a dynamic association between brain structure and function throughout neurodevelopment.

## Supporting Information

S1 File Hippocampal volume data. This contains all the raw data used in the analysis. This includes demographic, group status, brain volume, hippocampal volume and behavioural measures. Also included are a number of derived metrics included in the study, such as normalised volumes, longitudinal measure error and change in volume or behavioural measure over time. Not included are the values comprising the 3D hippocampal shape maps as these high dimensional data require specific software for visualisation and analysis. The authors will make these available on request, alongside the details of the necessary additional software packages.
(XLSX)

## References

[1] Botting N, Powls A, Cooke RW, Marlow N. Cognitive and educational outcome of very-low-birthweight children in early adolescence. Dev Med Child Neurol. 1998;40(10): 652–60. 9851233 10.1111/j.1469-8749.1998.tb12324.x

[2] Nosarti C, Giouroukou E, Healy E, Rifkin L, Walshe M, Reichenberg A, et al Grey and white matter distribution in very preterm adolescents mediates neurodevelopmental outcome. Brain. 2008;131(Pt 1): 205–17. 18056158 10.1093/brain/awm282

[3] Peterson BS, Anderson AW, Ehrenkranz R, Staib LH, Tageldin M, Colson E, et al Regional brain volumes and their later neurodevelopmental correlates in term and preterm infants. Pediatrics. 2003;111(5 Pt 1): 939–48. 12728069 10.1542/peds.111.5.939

[4] Indredavik MS, Vik T, Evensen KA, Skranes J, Taraldsen G, Brubakk AM. Perinatal risk and psychiatric outcome in adolescents born preterm with very low birth weight or term small for gestational age. J Dev Behav Pediatr. 2010;31(4): 286–94. 10.1097/DBP.0b013e3181d7b1d3 20431402

[5] Pavlova MA, Krageloh-Mann I. Limitations on the developing preterm brain: impact of periventricular white matter lesions on brain connectivity and cognition. Brain. 2013;136(Pt 4): 998–1011. 10.1093/brain/aws334 23550112

[6] Abernethy LJ, Palaniappan M, Cooke RW. Quantitative magnetic resonance imaging of the brain in survivors of very low birth weight. Arch Dis Child. 2002;87(4): 279–83. 12243993 10.1136/adc.87.4.279 PMC1763037

[7] Allin M, Matsumoto H, Santhouse AM, Nosarti C, AlAsady MH, Stewart AL, et al Cognitive and motor function and the size of the cerebellum in adolescents born very pre-term. Brain. 2001;124(Pt 1): 60–6. 11133787 10.1093/brain/124.1.60

[8] Hack M, Taylor HG. Perinatal brain injury in preterm infants and later neurobehavioral function. JAMA. 2000;284(15): 1973–4. 11035896 10.1001/jama.284.15.1973

[9] Nakamura Y, Nakashima T, Fukuda S, Nakashima H, Hashimoto T. Hypoxic-ischemic brain lesions found in asphyxiating neonates. Acta Pathol Jpn. 1986;36(4): 551–63. 3728012 10.1111/j.1440-1827.1986.tb01044.x

[10] McEwen BS. Plasticity of the hippocampus: adaptation to chronic stress and allostatic load. Ann N Y Acad Sci. 2001;933: 265–77. 12000027 10.1111/j.1749-6632.2001.tb05830.x

[11] Isaacs EB, Lucas A, Chong WK, Wood SJ, Johnson CL, Marshall C, et al Hippocampal volume and everyday memory in children of very low birth weight. Pediatr Res. 2000;47(6): 713–20. 10832727 10.1203/00006450-200006000-00006

[12] Lodygensky GA, Seghier ML, Warfield SK, Tolsa CB, Sizonenko S, Lazeyras F, et al Intrauterine growth restriction affects the preterm infant's hippocampus. Pediatr Res. 2008;63(4): 438–43. 10.1203/PDR.0b013e318165c005 18356754

[13] Beauchamp MH, Thompson DK, Howard K, Doyle LW, Egan GF, Inder TE, et al Preterm infant hippocampal volumes correlate with later working memory deficits. Brain. 2008;131(Pt 11): 2986–94. 10.1093/brain/awn227 18799516

[14] Thompson DK, Omizzolo C, Adamson C, Lee KJ, Stargatt R, Egan GF, et al Longitudinal growth and morphology of the hippocampus through childhood: Impact of prematurity and implications for memory and learning. Hum Brain Mapp. 2014;35(8): 4129–39. 10.1002/hbm.22464 24523026 PMC5516043

[15] Nosarti C, Al Asady MH, Frangou S, Stewart AL, Rifkin L, Murray RM. Adolescents who were born very preterm have decreased brain volumes. Brain. 2002;125(Pt 7): 1616–23. 12077010 10.1093/brain/awf157

[16] Thompson DK, Wood SJ, Doyle LW, Warfield SK, Lodygensky GA, Anderson PJ, et al Neonate hippocampal volumes: prematurity, perinatal predictors, and 2-year outcome. Ann Neurol. 2008;63(5): 642–51. 10.1002/ana.21367 18384167

[17] Thompson DK, Wood SJ, Doyle LW, Warfield SK, Egan GF, Inder TE. MR-determined hippocampal asymmetry in full-term and preterm neonates. Hippocampus. 2009;19(2): 118–23. 10.1002/hipo.20492 18767066 PMC2631622

[18] Andreasen NC, Flaum M, Swayze V 2nd, O'Leary DS, Alliger R, Cohen G, et al Intelligence and brain structure in normal individuals. Am J Psychiatry. 1993;150(1): 130–4. 8417555 10.1176/ajp.150.1.130

[19] Van Petten C. Relationship between hippocampal volume and memory ability in healthy individuals across the lifespan: review and meta-analysis. Neuropsychologia. 2004;42(10): 1394–413. 10.1016/j.neuropsychologia.2004.04.006 15193947

[20] Grunwald T, Kurthen M. Novelty detection and encoding for declarative memory within the human hippocampus. Clin EEG Neurosci. 2006;37(4): 309–14. 17073169 10.1177/155005940603700408

[21] Henke K, Weber B, Kneifel S, Wieser HG, Buck A. Human hippocampus associates information in memory. Proc Natl Acad Sci U S A. 1999;96(10): 5884–9. 10318979 10.1073/pnas.96.10.5884 PMC21955

[22] Maguire EA, Gadian DG, Johnsrude IS, Good CD, Ashburner J, Frackowiak RS, et al Navigation-related structural change in the hippocampi of taxi drivers. Proc Natl Acad Sci U S A. 2000;97(8): 4398–403. 10716738 10.1073/pnas.070039597 PMC18253

[23] Velakoulis D, Wood SJ, Wong MT, McGorry PD, Yung A, Phillips L, et al Hippocampal and amygdala volumes according to psychosis stage and diagnosis: a magnetic resonance imaging study of chronic schizophrenia, first-episode psychosis, and ultra-high-risk individuals. Arch Gen Psychiatry. 2006;63(2): 139–49. 10.1001/archpsyc.63.2.139 16461856

[24] Tupler LA, De Bellis MD. Segmented hippocampal volume in children and adolescents with posttraumatic stress disorder. Biol Psychiatry. 2006;59(6): 523–9. 10.1016/j.biopsych.2005.08.007 16199014

[25] Blumberg HP, Kaufman J, Martin A, Whiteman R, Zhang JH, Gore JC, et al Amygdala and hippocampal volumes in adolescents and adults with bipolar disorder. Arch Gen Psychiatry. 2003;60(12): 1201–8. 10.1001/archpsyc.60.12.1201 14662552

[26] Cole J, Toga AW, Hojatkashani C, Thompson P, Costafreda SG, Cleare AJ, et al Subregional hippocampal deformations in major depressive disorder. J Affect Disord. 2010;126(1–2): 272–7. 10.1016/j.jad.2010.03.004 20392498 PMC3197834

[27] Buehlmann E, Berger GE, Aston J, Gschwandtner U, Pflueger MO, Borgwardt SJ, et al Hippocampus abnormalities in at risk mental states for psychosis? A cross-sectional high resolution region of interest magnetic resonance imaging study. J Psychiatr Res. 2010;44(7): 447–53. 10.1016/j.jpsychires.2009.10.008 19939408

[28] Ho BC, Magnotta V. H ippocampal volume deficits and shape deformities in young biological relatives of schizophrenia probands. Neuroimage. 2010;49(4): 3385–93. 10.1016/j.neuroimage.2009.11.033 19941961 PMC2818551

[29] Gimenez M, Junque C, Narberhaus A, Caldu X, Salgado-Pineda P, Bargallo N, et al Hippocampal gray matter reduction associates with memory deficits in adolescents with history of prematurity. NeuroImage. 2004;23(3): 869–77. 15528087 10.1016/j.neuroimage.2004.07.029

[30] Rogers CE, Anderson PJ, Thompson DK, Kidokoro H, Wallendorf M, Treyvaud K, et al Regional cerebral development at term relates to school-age social-emotional development in very preterm children. J Am Acad Child Adolesc Psychiatry. 2012;51(2): 181–91. 10.1016/j.jaac.2011.11.009 22265364 PMC3411187

[31] Anderson P, Doyle LW. Neurobehavioral outcomes of school-age children born extremely low birth weight or very preterm in the 1990s. JAMA. 2003;289(24): 3264–72. 12824207 10.1001/jama.289.24.3264

[32] Botting N, Powls A, Cooke RW, Marlow N. Attention deficit hyperactivity disorders and other psychiatric outcomes in very low birthweight children at 12 years. J Child Psychol Psychiatry. 1997;38(8): 931–41. 9413793 10.1111/j.1469-7610.1997.tb01612.x

[33] Johnson S, Hollis C, Kochhar P, Hennessy E, Wolke D, Marlow N. Psychiatric disorders in extremely preterm children: longitudinal finding at age 11 years in the EPICure study. J Am Acad Child Adolesc Psychiatry. 2010;49(5): 453–63 e1. 20431465

[34] Limperopoulos C, Bassan H, Sullivan NR, Soul JS, Robertson RL Jr., Moore M, et al Positive screening for autism in ex-preterm infants: prevalence and risk factors. Pediatrics. 2008;121(4): 758–65. 10.1542/peds.2007-2158 18381541 PMC2703587

[35] Taylor HG, Klein N, Minich NM, Hack M. Middle-school-age outcomes in children with very low birthweight. Child Dev. 2000;71(6): 1495–511. 11194251 10.1111/1467-8624.00242

[36] Nosarti C, Murray RM, Reichenberg A, Cnattingius S, Lambe MP, Yin L, et al Preterm birth and psychiatric disorders in young adult life. Arch Gen Psychiatry. 2012;69(6): 610–7. 10.1001/archgenpsychiatry.2011.1374 22660967

[37] Raikkonen K, Pesonen AK, Heinonen K, Kajantie E, Hovi P, Jarvenpaa AL, et al Depression in young adults with very low birth weight: the Helsinki study of very low-birth-weight adults. Arch Gen Psychiatry. 2008;65(3): 290–6. 10.1001/archgenpsychiatry.2007.40 18316675

[38] Geddes JR, Verdoux H, Takei N, Lawrie SM, Bovet P, Eagles JM, et al Schizophrenia and complications of pregnancy and labor: an individual patient data meta-analysis. Schizophr Bull. 1999;25(3): 413–23. 10478777 10.1093/oxfordjournals.schbul.a033389

[39] Arnold SE, Trojanowski JQ. Human fetal hippocampal development: I. Cytoarchitecture, myeloarchitecture, and neuronal morphologic features. J Comp Neurol. 1996;367(2): 274–92. 10.1002/(SICI)1096-9861 8708010

[40] Benes FM, Turtle M, Khan Y, Farol P. Myelination of a key relay zone in the hippocampal formation occurs in the human brain during childhood, adolescence, and adulthood. Arch Gen Psychiatry. 1994;51(6): 477–84. 8192550 10.1001/archpsyc.1994.03950060041004

[41] Gogtay N, Nugent TF 3rd, Herman DH, Ordonez A, Greenstein D, Hayashi KM, et al Dynamic mapping of normal human hippocampal development. Hippocampus. 2006;16(8): 664–72. 10.1002/hipo.20193 16826559

[42] Ment LR, Kesler S, Vohr B, Katz KH, Baumgartner H, Schneider KC, et al Longitudinal brain volume changes in preterm and term control subjects during late childhood and adolescence. Pediatrics. 2009;123(2): 503–11. 10.1542/peds.2008-0025 19171615 PMC2679898

[43] Parker J, Mitchell A, Kalpakidou A, Walshe M, Jung HY, Nosarti C, et al Cerebellar growth and behavioural & neuropsychological outcome in preterm adolescents. Brain. 2008;131(Pt 5): 1344–51. 18372312 10.1093/brain/awn062

[44] Allin M, Nosarti C, Narberhaus A, Walshe M, Frearson S, Kalpakidou A, et al Growth of the corpus callosum in adolescents born preterm. Arch Pediatr Adolesc Med. 2007;161(12): 1183–9. 18056564 10.1001/archpedi.161.12.1183

[45] Shaw P, Greenstein D, Lerch J, Clasen L, Lenroot R, Gogtay N, et al Intellectual ability and cortical development in children and adolescents. Nature. 2006;440(7084): 676–9. 16572172 10.1038/nature04513

[46] Gogtay N, Thompson PM. Mapping gray matter development: implications for typical development and vulnerability to psychopathology. Brain Cogn. 2010;72(1): 6–15. 10.1016/j.bandc.2009.08.009 19796863 PMC2815268

[47] Barbas H, Blatt GJ. Topographically specific hippocampal projections target functionally distinct prefrontal areas in the rhesus monkey. Hippocampus. 1995;5(6): 511–33. 10.1002/hipo.450050604 8646279

[48] Strange B, Dolan R. Functional segregation within the human hippocampus. Mol Psychiatry. 1999;4(6): 508–11. 10578231 10.1038/sj.mp.4000593

[49] Phillips ML, Drevets WC, Rauch SL, Lane R. Neurobiology of emotion perception II: Implications for major psychiatric disorders. Biol Psychiatry. 2003;54(5): 515–28. 12946880 10.1016/s0006-3223(03)00171-9

[50] Thompson PM, Hayashi KM, De Zubicaray GI, Janke AL, Rose SE, Semple J, et al Mapping hippocampal and ventricular change in Alzheimer disease. Neuroimage. 2004;22(4): 1754–66. 10.1016/j.neuroimage.2004.03.040 15275931

[51] Desgranges B, Baron JC, Eustache F. The functional neuroanatomy of episodic memory: the role of the frontal lobes, the hippocampal formation, and other areas. Neuroimage. 1998;8(2): 198–213. 10.1006/nimg.1998.0359 9740762

[52] van Groen T, Wyss JM. Extrinsic projections from area CA1 of the rat hippocampus: olfactory, cortical, subcortical, and bilateral hippocampal formation projections. J Comp Neurol. 1990;302(3): 515–28. 10.1002/cne.903020308 1702115

[53] Brugger P, Dowdy MA, Graves RE. From superstitious behavior to delusional thinking: the role of the hippocampus in misattributions of causality. Med Hypotheses. 1994;43(6): 397–402. 7739412 10.1016/0306-9877(94)90015-9

[54] Stewart AL, Costello AM, Hamilton PA, Baudin J, Townsend J, Bradford BC, et al Relationship between neurodevelopmental status of very preterm infants at one and four years. Dev Med Child Neurol. 1989;31(6): 756–65. 2599269 10.1111/j.1469-8749.1989.tb04071.x

[55] Costello AM, Hamilton PA, Baudin J, Townsend J, Bradford BC, Stewart AL, et al Prediction of neurodevelopmental impairment at four years from brain ultrasound appearance of very preterm infants. Dev Med Child Neurol. 1988;30(6): 711–22. 2466720 10.1111/j.1469-8749.1988.tb14633.x

[56] Roth SC, Baudin J, McCormick DC, Edwards AD, Townsend J, Stewart AL, et al Relation between ultrasound appearance of the brain of very preterm infants and neurodevelopmental impairment at eight years. Dev Med Child Neurol. 1993;35(9): 755–68. 7689065 10.1111/j.1469-8749.1993.tb11727.x

[57] Rushe T, Temple C, Rifkin L, Woodruff P, Bullmore E, Stewart A, et al Lateralisation of language function in young adults born very preterm. Arch Dis Child Fetal Neonatal Ed. 2004;89(2): F112–F8. 10.1136/adc.2001.005314 14977893 PMC1756037

[58] Rutter M. Classification and categorization in child psychiatry. Journal of Child Psychology and Psychiatry. 1965;6(2): 71–83. 5851100 10.1111/j.1469-7610.1965.tb02229.x

[59] Connell HM, Irvine L, Rodney J. The prevalence of psychiatric disorder in rural school children. Australia and New Zealand Journal of Psychiatry. 1982;16(2): 43–6. 6982040 10.3109/00048678209161190

[60] Rutter M, Tizard J, Whitmore K. Education, Health and Behaviour London: Longman; 1970.

[61] Peters E, Joseph S, Day S, Garety P. Measuring delusional ideation: the 21-item Peters et al. Delusions Inventory (PDI). Schizophr Bull. 2004;30(4): 1005–22. 15954204 10.1093/oxfordjournals.schbul.a007116

[62] Preti A, Rocchi MB, Sisti D, Mura T, Manca S, Siddi S, et al The psychometric discriminative properties of the Peters et al Delusions Inventory: a receiver operating characteristic curve analysis. Compr Psychiatry. 2007;48(1): 62–9. 10.1016/j.comppsych.2006.05.003 17145284

[63] Goldberg D, Williams P. A user's guide to the General Health Questionnaire Windsor, UK: NFER-Nelson; 1988.

[64] Goldberg DP, Gater R, Sartorius N, Ustun TB, Piccinelli M, Gureje O, et al The validity of two versions of the GHQ in the WHO study of mental illness in general health care. Psychol Med. 1997;27(1): 191–7. 9122299 10.1017/s0033291796004242

[65] Narr KL, Thompson PM, Szeszko P, Robinson D, Jang S, Woods RP, et al Regional specificity of hippocampal volume reductions in first-episode schizophrenia. Neuroimage. 2004;21(4): 1563–75. 10.1016/j.neuroimage.2003.11.011 15050580

[66] Woods RP. Multitracer: a Java-based tool for anatomic delineation of grayscale volumetric images. Neuroimage. 2003;19(4): 1829–34. 12948737 10.1016/s1053-8119(03)00243-x

[67] Shi Y, Morra JH, Thompson PM, Toga AW. Inverse-consistent surface mapping with Laplace-Beltrami eigen-features. Inf Process Med Imaging. 2009;21: 467–78. 19694286 10.1007/978-3-642-02498-6_39 PMC2970526

[68] Rex DE, Ma JQ, Toga AW. The LONI Pipeline Processing Environment. Neuroimage. 2003;19(3): 1033–48. 12880830 10.1016/s1053-8119(03)00185-x

[69] Duvernoy H. The human hippocampus An Atlas of Applied Anatomy. Munich: J.F. Bergmann Verlag; 1988.

[70] Stein JL, Medland SE, Vasquez AA, Hibar DP, Senstad RE, Winkler AM, et al Identification of common variants associated with human hippocampal and intracranial volumes. Nat Genet. 2012;44(5): 552–61. 10.1038/ng.2250 22504417 PMC3635491

[71] Reig S, Moreno C, Moreno D, Burdalo M, Janssen J, Parellada M, et al Progression of brain volume changes in adolescent-onset psychosis. Schizophr Bull. 2009;35(1): 233–43. 10.1093/schbul/sbm160 18222929 PMC2643965

[72] Miller GA, Chapman JP. Misunderstanding analysis of covariance. J Abnorm Psychol. 2001;110(1): 40–8. 11261398 10.1037//0021-843x.110.1.40

[73] Benjamini Y, Hochberg Y. Controlling the False Discovery Rate—a Practical and Powerful Approach to Multiple Testing. J Roy Stat Soc B Met. 1995;57(1): 289–300.

[74] Nosarti C, Allin MP, Frangou S, Rifkin L, Murray RM. Hyperactivity in adolescents born very preterm is associated with decreased caudate volume. Biol Psychiatry. 2005;57(6): 661–6. 15780854 10.1016/j.biopsych.2004.12.003

[75] Van Essen DC. A tension-based theory of morphogenesis and compact wiring in the central nervous system. Nature. 1997;385(6614): 313–8. 10.1038/385313a0 9002514

[76] Lubsen J, Vohr B, Myers E, Hampson M, Lacadie C, Schneider KC, et al Microstructural and functional connectivity in the developing preterm brain. Seminars in perinatology. 2011;35(1): 34–43. 10.1053/j.semperi.2010.10.006 21255705 PMC3063450

[77] Bigler ED, Blatter DD, Anderson CV, Johnson SC, Gale SD, Hopkins RO, et al Hippocampal volume in normal aging and traumatic brain injury. AJNR Am J Neuroradiol. 1997;18(1): 11–23. 9010515 PMC8337859

[78] Mattai A, Hosanagar A, Weisinger B, Greenstein D, Stidd R, Clasen L, et al Hippocampal volume development in healthy siblings of childhood-onset schizophrenia patients. Am J Psychiatry. 2011;168(4): 427–35. 10.1176/appi.ajp.2010.10050681 21245087 PMC3289129

[79] Erickson KI, Voss MW, Prakash RS, Basak C, Szabo A, Chaddock L, et al Exercise training increases size of hippocampus and improves memory. Proc Natl Acad Sci U S A. 2011;108(7): 3017–22. 10.1073/pnas.1015950108 21282661 PMC3041121

[80] Eisenberger NI, Gable SL, Lieberman MD. F unctional magnetic resonance imaging responses relate to differences in real-world social experience. Emotion. 2007;7(4): 745–54. 10.1037/1528-3542.7.4.745 18039043

[81] St Jacques PL, Botzung A, Miles A, Rubin DC. Functional neuroimaging of emotionally intense autobiographical memories in post-traumatic stress disorder. J Psychiatr Res. 2011;45(5): 630–7. 10.1016/j.jpsychires.2010.10.011 21109253 PMC3081954

[82] Rugg MD, Otten LJ, Henson RN. The neural basis of episodic memory: evidence from functional neuroimaging. Philos Trans R Soc Lond B Biol Sci. 2002;357(1424): 1097–110. 12217177 10.1098/rstb.2002.1102 PMC1693015

[83] Dagnall N, Munley G, Parker A. Memory aberrations, transliminality, and delusional ideation. Percept Mot Skills. 2008;106(1): 67–75. 18459357 10.2466/pms.106.1.67-75

[84] Linney YM, Peters ER, Ayton P. Reasoning biases in delusion-prone individuals. Br J Clin Psychol. 1998;37 (Pt 3): 285–302. 9784884 10.1111/j.2044-8260.1998.tb01386.x

[85] Broome MR, Johns LC, Valli I, Woolley JB, Tabraham P, Brett C, et al Delusion formation and reasoning biases in those at clinical high risk for psychosis. Br J Psychiatry. 2007;51: s38–42. 10.1192/bjp.191.51.s38 18055936

[86] Broome MR, Day F, Valli I, Valmaggia L, Johns LC, Howes O, et al Delusional ideation, manic symptomatology and working memory in a cohort at clinical high-risk for psychosis: a longitudinal study. Europ Psychiatry. 2012;27(4): 258–63. 10.1016/j.eurpsy.2010.07.008 20934858

[87] Baddeley A, Jarrold C, Vargha-Khadem F. Working memory and the hippocampus. J Cog Neurosci. 2011;23(12): 3855–61. 10.1162/jocn_a_00066 21671734

[88] Velakoulis D, Pantelis C, McGorry PD, Dudgeon P, Brewer W, Cook M, et al Hippocampal volume in first-episode psychoses and chronic schizophrenia: a high-resolution magnetic resonance imaging study. Arch Gen Psychiatry. 1999;56(2): 133–41. 10025437 10.1001/archpsyc.56.2.133

[89] Groen W, Teluij M, Buitelaar J, Tendolkar I. Amygdala and hippocampus enlargement during adolescence in autism. J Am Acad Child Adolesc Psychiatry. 2010;49(6): 552–60. 10.1016/j.jaac.2009.12.023 20494265

[90] Plessen KJ, Bansal R, Zhu H, Whiteman R, Amat J, Quackenbush GA, et al Hippocampus and amygdala morphology in attention-deficit/hyperactivity disorder. Arch Gen Psychiatry. 2006;63(7): 795–807. 10.1001/archpsyc.63.7.795 16818869 PMC2367150

[91] Weinberger DR, Berman KF, Suddath R, Torrey EF. Evidence of dysfunction of a prefrontal-limbic network in schizophrenia: a magnetic resonance imaging and regional cerebral blood flow study of discordant monozygotic twins. Am J Psychiatry. 1992;149(7): 890–7. 1609867 10.1176/ajp.149.7.890

[92] Csernansky JG, Joshi S, Wang L, Haller JW, Gado M, Miller JP, et al Hippocampal morphometry in schizophrenia by high dimensional brain mapping. Proc Natl Acad Sci U S A. 1998;95(19): 11406–11. 9736749 10.1073/pnas.95.19.11406 PMC21655

[93] Adriano F, Caltagirone C, Spalletta G. Hippocampal volume reduction in first-episode and chronic schizophrenia: a review and meta-analysis. The Neuroscientist. 2012;18(2): 180–200. 10.1177/1073858410395147 21531988

[94] Rosoklija G, Toomayan G, Ellis SP, Keilp J, Mann JJ, Latov N, et al Structural abnormalities of subicular dendrites in subjects with schizophrenia and mood disorders: preliminary findings. Arch Gen Psychiatry. 2000;57(4): 349–56. 10768696 10.1001/archpsyc.57.4.349

[95] Jung HY, Chang JS, Yi JS, Hwang S, Shin HK, Kim JH, et al Measuring psychosis proneness in a nonclinical Korean population: is the Peters et al Delusions Inventory useful for assessing high-risk individuals? Compr Psychiatry. 2008;49(2): 202–10. 10.1016/j.comppsych.2007.08.011 18243895

[96] Shaw P, Gogtay N, Rapoport J. Childhood psychiatric disorders as anomalies in neurodevelopmental trajectories. Hum Brain Mapp. 2010;31(6): 917–25. 10.1002/hbm.21028 20496382 PMC6870870

